# Numerical investigations of an ultra-compact polarization beam splitter based on augmented low-index guiding and subwavelength grating structures

**DOI:** 10.1038/s41598-018-35841-2

**Published:** 2018-11-26

**Authors:** Chia-Chien Huang

**Affiliations:** Department of Physics and Institute of Nanoscience, National Chung Hsing University, 145, Xingda Rd., Taichung, 402 Taiwan Republic of China

## Abstract

We report the design of an ultra-compact polarization beam splitter with high performance that is based on augmented low-index guiding and subwavelength grating (SWG) structures. The transverse-electric (TE) and transverse-magnetic (TM) modes are confined in high-index silicon (Si) and low-index silicon nitride (Si_3_N_4_), respectively. They are separated by using, respectively, a gradually curved Si waveguide and a Si_3_N_4_ SWG structure with optimal grating-element. The footprint of the proposed polarization beam splitters (PBS) is 2.9 × 2.25 μm^2^. The device offers high polarization extinction ratios (PERs) of ~18 dB for the two polarizations, with low insertion losses of ~0.22 dB (~0.71 dB) for the TE (TM) mode at the wavelength of 1550 nm. Over the broad band from *λ* = 1500–1650 nm, the PERs of the TE and TM modes are above 17 and 16 dB, respectively. By narrowing the operating band to the range from *λ* = 1500 to 1600 nm, the proposed PBS provides PERs of **>**17 dB for both polarizations. Finally, the fabrication tolerance of the designed PBS is also addressed and discussed in detail.

## Introduction

To achieve the high transmission demands of optical communication systems and construct photonic integrated circuits, devices such as polarization beam splitters (PBSs) and polarization rotators play pivotal roles in manipulating optical signals. Accordingly, considerable effort has been devoted to such components over the years^1–3^. In the present work, we focus on a PBS, the function of which is to separate the transverse-electric (TE) and transverse-magnetic (TM) polarizations into different waveguides. Important criteria for assessing a PBS include the device footprint, polarization extinction ratio (PER), insertion loss (IL), operating bandwidth, and fabrication tolerance. Many types of PBSs^4–12^ have been reported, based on various mechanisms: adiabatic mode-evolution devices (AMEs)^4^, directional couplers (DCs)^5–8^, and multimode interference structures (MMIs)^9–12^. For compatibility with mature semiconductor fabrication techniques, most PBSs have adopted silicon-on-insulator (SOI) platforms to decrease the device sizes by utilizing high-index contrast materials. The AME-based PBSs^4^ are very long (~200 μm), due to their slowly evolving geometries, but they have better fabrication tolerance and broadband operation. In contrast, DC-based PBSs^5–8^—which separate one selected mode by coupling it to the cross bar while the remaining mode propagates along the bar—are several to tens of micrometers long and provide practical PERs of 10–20 dB. However, their operating bandwidths are narrow, due to the requirement of utilizing phase-matched modes with precisely tuned couplings. The MMI-based PBSs^9–12^ employ a simpler fabrication process and have greater fabrication tolerance; however, the dimensions of conventional MMI devices^13,14^ are determined by the common multiple of the self-imaging lengths^15^ of the TE and TM modes, resulting in long devices. Some innovative designs have recently been reported for shortening the lengths of MMI-based PBSs. These include metal–insulator–metal (MIM)-embedded^9^ (∼44 μm) and hybrid-plasmonic-waveguide (HPW)^11^ (∼2.5 μm) MMIs, in which surface plasmon polariton (SPP)-guided modes are excited^16^ and for which most of the electric field is perpendicular to the metal surface. Although plasmonic-based PBSs can effectively shrink the dimensions, the inherent ohmic losses of the SPP modes are much larger than those of dielectric-guided modes. In addition, there must be input and output beams to couple into and out of the plasmonic-based PBSs, because the mode distributions in dielectric waveguides or optical fibers are incompatible with SPP modes^17^. The performance obtained is therefore influenced by the input and output coupling efficiencies.

Recently, the augmented low-index guiding (ALIG) mechanism^18^ has been utilized to construct a PBS. An ALIG waveguide consists of a two-layer system with a high-contrast index. It can achieve high power confinement in a low-index Si_3_N_4_ layer by appropriately choosing the thickness of a high-index Si layer. Based on the ALIG structure, the investigators reported numerically^19^ and experimentally^20^ a PBS with the dimensions of 4.8 μm (length) × 1.6 μm (width). They adopted an asymmetric MMI section made of Si to couple the TE mode to the cross port and a Si_3_N_4_ waveguide to guide the TM mode directly to the bar port. However, the PER of the TM mode is relatively low due to the excitation of a higher-order TM mode (TM_1_) in the MMI structure. The length of this PBS is still too long, due to the requirement of forming a mirror image in the MMI section^15^. Recently, Halir *et al*.^21,22^ have employed subwavelength gratings (SWGs), optimally adjusting the grating period and duty cycle to achieve a threefold reduction in the beat length of the MMI structure, compared to conventional MMI structures, by utilizing the structural anisotropy of the grating structure. For the design of photonic devices, SWGs offer an additional degree of freedom for achieving the desired properties. Many functional devices based on SWGs have therefore been reported, including PBSs^23,24^, polarization splitter-rotators^25^, polarization-independent DCs^26^, polarization-insensitive power splitters^27^, and contra-directional couplers^28^. Each has achieved reasonable performance and has a smaller footprint compared to conventional structures.

In this work, we propose a PBS that employs an ALIG structure as the input port. Rather than an MMI structure^19,20^, we use a curved Si waveguide and a SWG Si_3_N_4_ waveguide to separate the TE and TM modes, respectively. Consequently, the length of our PBS device can be effectively shrunk to 2.9 μm while maintaining satisfactory performance and broadband operating capability. In particular, the PERs of the two modes can be enhanced up to about 18 dB at the wavelength of 1.55 μm by optimally adjusting the duty cycle of the grating structure. The entire footprint of the proposed PBS is about 2.9 μm long × 2.25 μm wide. The materials used here are CMOS-compatible, making the fabrication requirements easier.

## Results

### Mode properties of the designed PBS

**Figure 1:**
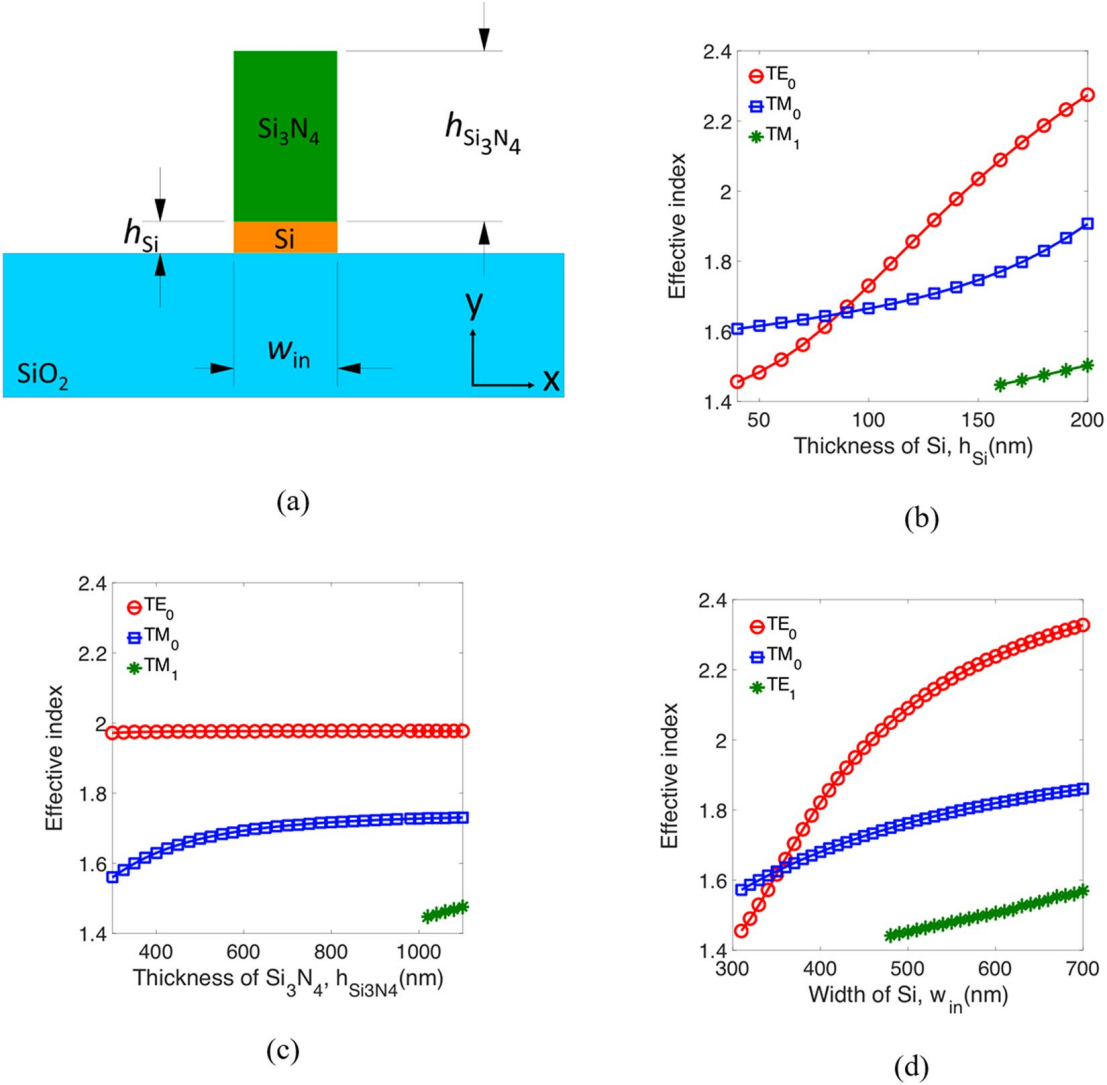
(**a**) A cross section of the ALIG structure. Effective index versus (**b**) the thickness of Si, *h*_*Si*_, (**c**) the thickness of Si_3_N_4_, *h*_Si3N4_, and (**d**) the width of Si, *w*_in_.

To design the proposed PBS, we first analyze the mode characteristics of an ALIG waveguide. A cross section of such a waveguide is shown in Fig. 1(a); it is formed by depositing silicon nitride (Si_3_N_4_) on a SOI platform and covering it with a low-index cladding (here, we choose air). The widths of the Si and Si_3_N_4_ are both *w*_in_, and the heights of the Si and Si_3_N_4_ layers are *h*_Si_ and *h*_Si3N4_, respectively. The refractive indices^29^ of the Si, Si_3_N_4_, and SiO_2_ we used are *n*_*Si*_ = 3.4777, *n*_*Si3N4*_ = 1.9963, and *n*_*SiO2*_ = 1.4440, respectively, and the operating wavelength of the device is *λ* = 1.55 μm. According to the guiding principle for an ALIG structure^18^, the Si layer must be thin enough to push most of the power of the TM mode (for which most of the electric field is in the *y* direction) into the Si_3_N_4_ layer. On other hand, the high-index Si layer is used as a conventional waveguide to confine the TE mode (for which most of the electric field is in the *x* direction). Using the boundary-mode analysis in the commercial COMSOL Multiphysics software, we have analyzed the mode characteristics versus the waveguide dimensions *h*_Si_, *h*_Si3N4_, and *w*_in_, as shown in Fig. 1(b–d), respectively. From the calculated results, we find that the geometrical conditions *h*_Si_ < 160 nm, *h*_Si3N4_ < 1000 nm, and *w*_in_ < 480 nm support only the fundamental TE and TM modes (TE_0_ and TM_0_).

**Figure 2:**
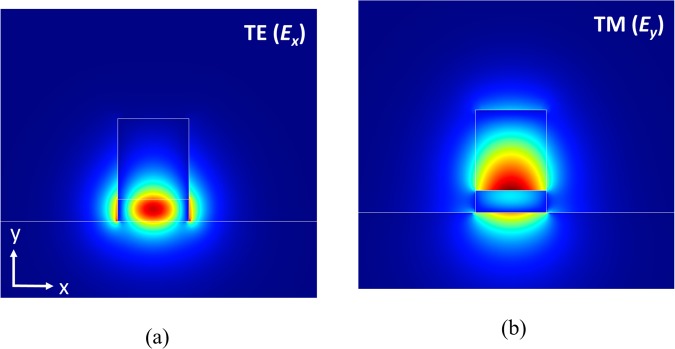
The mode fields of (**a**) the TE (*E*_*x*_) and (**b**) the TM (*E*_*y*_) modes of an ALIG structure with the dimensions *h*_Si_ = 140 nm, *h*_Si3N4_ = 650 nm and *w*_in_ = 450 nm.

Considering the experimental feasibility for a conventional full-etch fabrication technique, we therefore chose the dimensions *h*_Si_ = 140 nm, *h*_Si3N4_ = 650 nm, and *w*_in_ = 450 nm for the input port, although choosing larger *h*_Si3N4_ can attain higher PER. The mode profiles of the fundamental TE (*E*_*x*_) and TM (*E*_*y*_) modes are shown in Fig. 2(a,b), respectively, and the calculated effective indices for the two modes are *n*_TE_ = 1.9763 and *n*_TM_ = 1.6725. Unlike the hybrid plasmonic modes^17^, the ALIG structure supports pure dielectric modes, which eliminates the high ohmic losses in a metal. Clearly, most of the TE and TM mode fields are concentrated in the Si and Si_3_N_4_ regions, respectively. For the TE mode profile shown in Fig. 2(a), the power fractions in the Si and Si_3_N_4_ regions are about 53.4% and 25.4%, respectively. In contrast, for the TM mode profile, the corresponding power fractions in the Si and Si_3_N_4_ regions are about 11.7% and 63.9%, respectively, as shown in Fig. 2(b). These calculated results demonstrate that most of the power of the TM mode can indeed be confined in the low-index Si_3_N_4_ region through a proper choice of the thickness of the high-index Si layer. We have used the geometrical dimensions mentioned above to design the proposed PBS described below, unless stated otherwise.

### Propagating performance of the designed PBS

**Figure 3:**
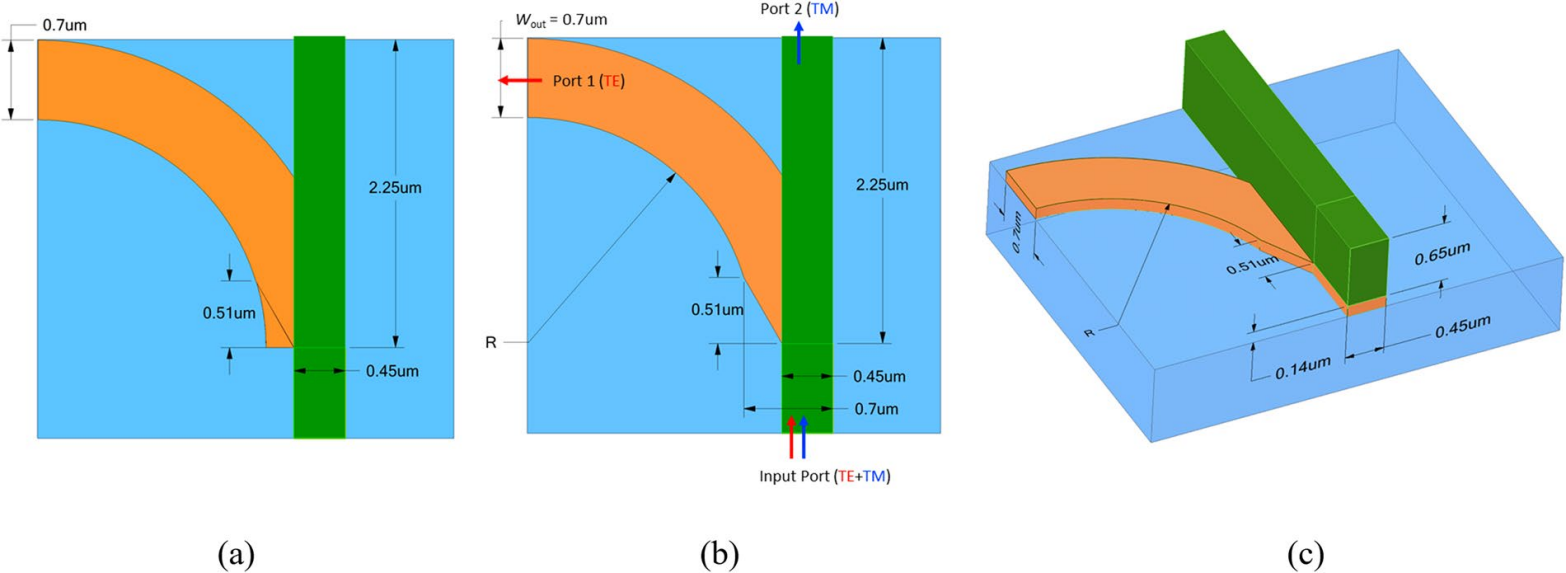
(**a**) Top view of the proposed PBS, in which the bottom curved silicon strip (orange part) is composed of a 90° bent structure. (**b**) the same as (**a**), except tapering input part of the bent silicon strip. (**c**) 3D schematic diagram.

Using the ALIG structure as the input waveguide, we designed a PBS according to the criteria mentioned previously. Figure 3(a) shows the top view of the proposed PBS consisted of two parts. One is a 90° bent Si strip waveguide, which guides the TE mode. It has radius of curvature *R* and is formed on a SiO_2_ substrate. The other part is a Si_3_N_4_ strip waveguide, which guides the TM mode and which is deposited on top of the Si waveguide. The detailed fabricated process of the proposed PBS is not shown here due to the similar process as shown in the previous paper^20^. The design processes are as follows. First, to reduce the bending loss, we design a wider silicon strip of 700 nm while still operating in the fundamental mode for the TE output port. Second, making TE power undergo a smooth transition to reduce the input scattering, we taper the width of input port of the bent Si strip from 700 nm to 450 nm to conform the width of the input ALIG structure (shown in Fig. 1(a)) as shown in Fig. 3(b). Finally, choosing a radius of curvature *R* by optimizing the performances. The cladding here is air, and the 3D schematic diagram of the proposed PBS is shown in Fig. 3(c). The thickness of the Si layer is *h*_Si_ = 140 nm, and its width at the TE output port is *w*_out_ = 700 nm but is 450 nm at the input port after tapering. The thickness of the Si_3_N_4_ is *h*_Si3N4_ = 650 nm.

**Figure 4:**
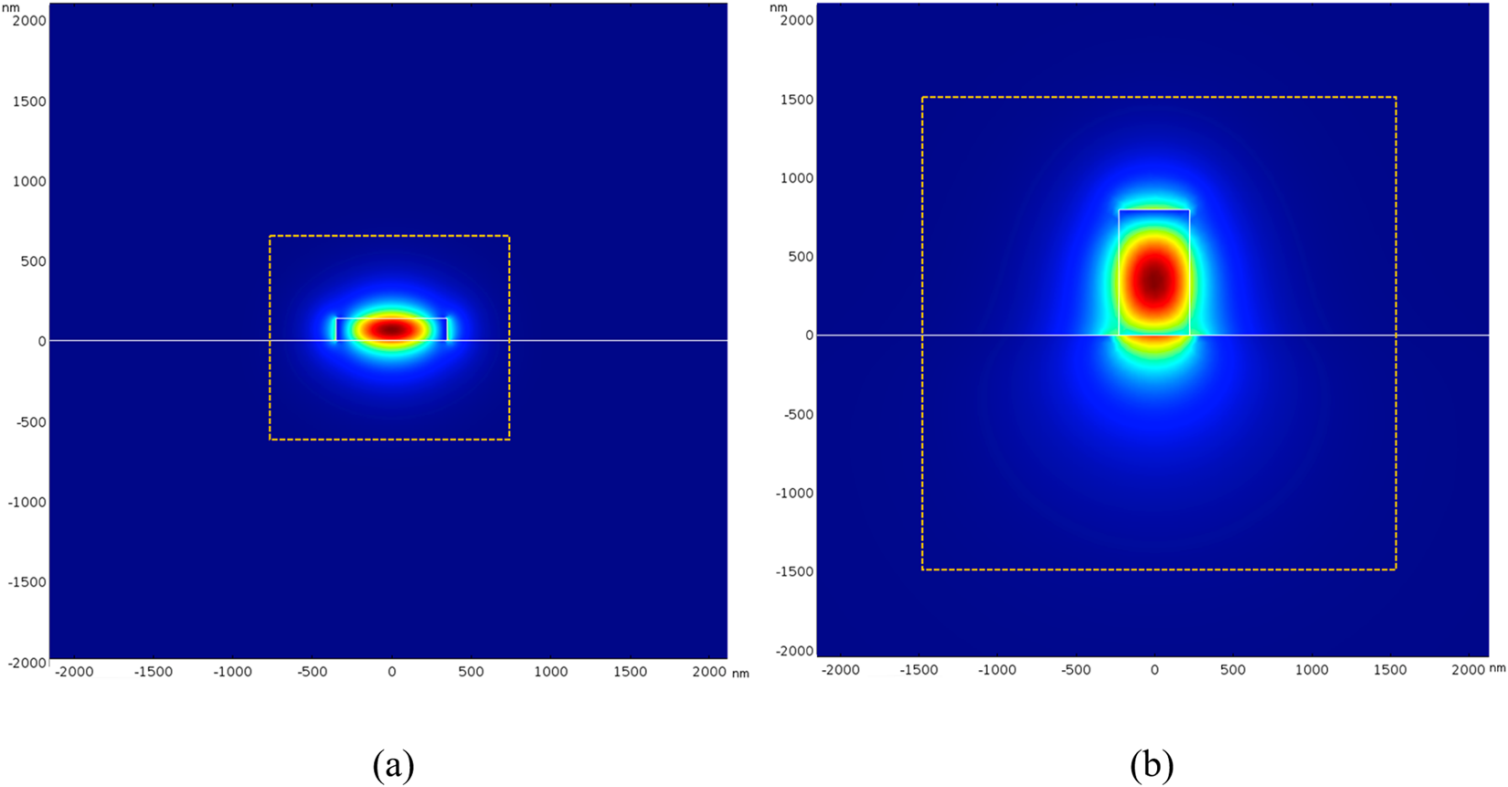
Mode fields of (**a**) the TE (*E*_*x*_) and (**b**) the TM (*E*_*y*_) modes at the output ports. The yellow dashed lines indicate the regions for computing the output powers.

Before analyzing the performance of the proposed PBS, we show the mode distributions of the TE and TM modes at the output ports in Fig. 4(a,b), respectively, for comparison with the mode distributions at the input port. The yellow dashed lines indicate the regions of TE and TM output ports for computing the output powers. The yellow rectangular boundaries represent the amplitude of electric fields decaying to 1% of their peak values. The calculated effective indices of the two modes are *n*_TE_ = 2.1904 and *n*_TM_ = 1.5282. For the TE mode profile, the power fraction in the Si region is about 62.4%, and that in the Si_3_N_4_ region is about 71.0% for the TM mode profile. Clearly, the overlap of the TE mode profiles between the input port, as shown in Fig. 2(a) above, and the output port is very high, resulting in high transmission of the TE power. In contrast, the TM mode profile at the input port increases moderately to become that at the output port. Consequently, the transmission is a little lower than that of the TE mode.

**Figure 5:**
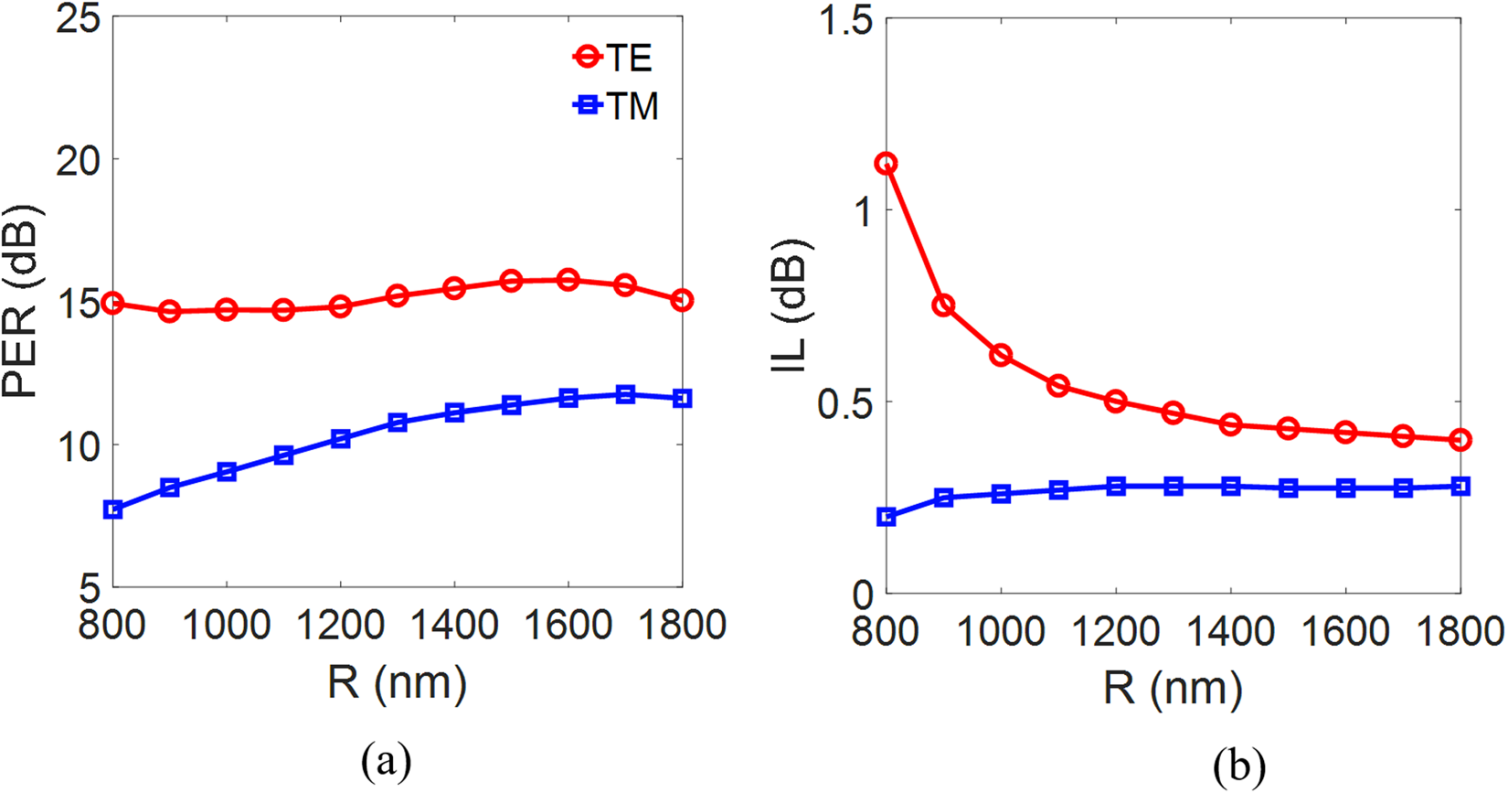
(**a**) The PER and (**b**) the IL of the TE and TM modes *versus* the radius of curvature *R* of the curved Si waveguide.

To evaluate the performance of the proposed PBS quantitatively, we have studied the PER and IL of the two modes (as defined in the method Section below). The calculated values of PER and IL versus the radius of curvature *R* of the curved Si waveguide are shown in Fig. 5(a,b), respectively. The calculated PERs (ILs) of the TE and TM modes are around 15.35 dB (0.48 dB) and 11.11 dB (0.28 dB), respectively, for *R* > 1550 nm. We note that, as expected, the IL of the TM mode is not much influenced by the varying *R* of the curved Si waveguide, but the IL of the TE mode increases significantly when *R* is smaller than 1440 nm. The PER of the TE mode is around 15 dB, varying slightly with *R*. This is because the coupling of power from the TM mode to the curved Si waveguide decreases as *R* decreases, compensating for the greater loss of TE power at smaller *R*. In contrast, the PER of the TM mode decreases as *R* decreases because of the greater bending loss of the TE mode. Although we can obtain satisfactory ILs for the two modes at the condition of *R* > 1550 nm, the PER of the TM mode remains merely at an unsatisfactory level. Therefore, there is a sufficiently large space to improve the PER of the TM mode, improving the whole performance of the proposed PBS.

**Figure 6:**
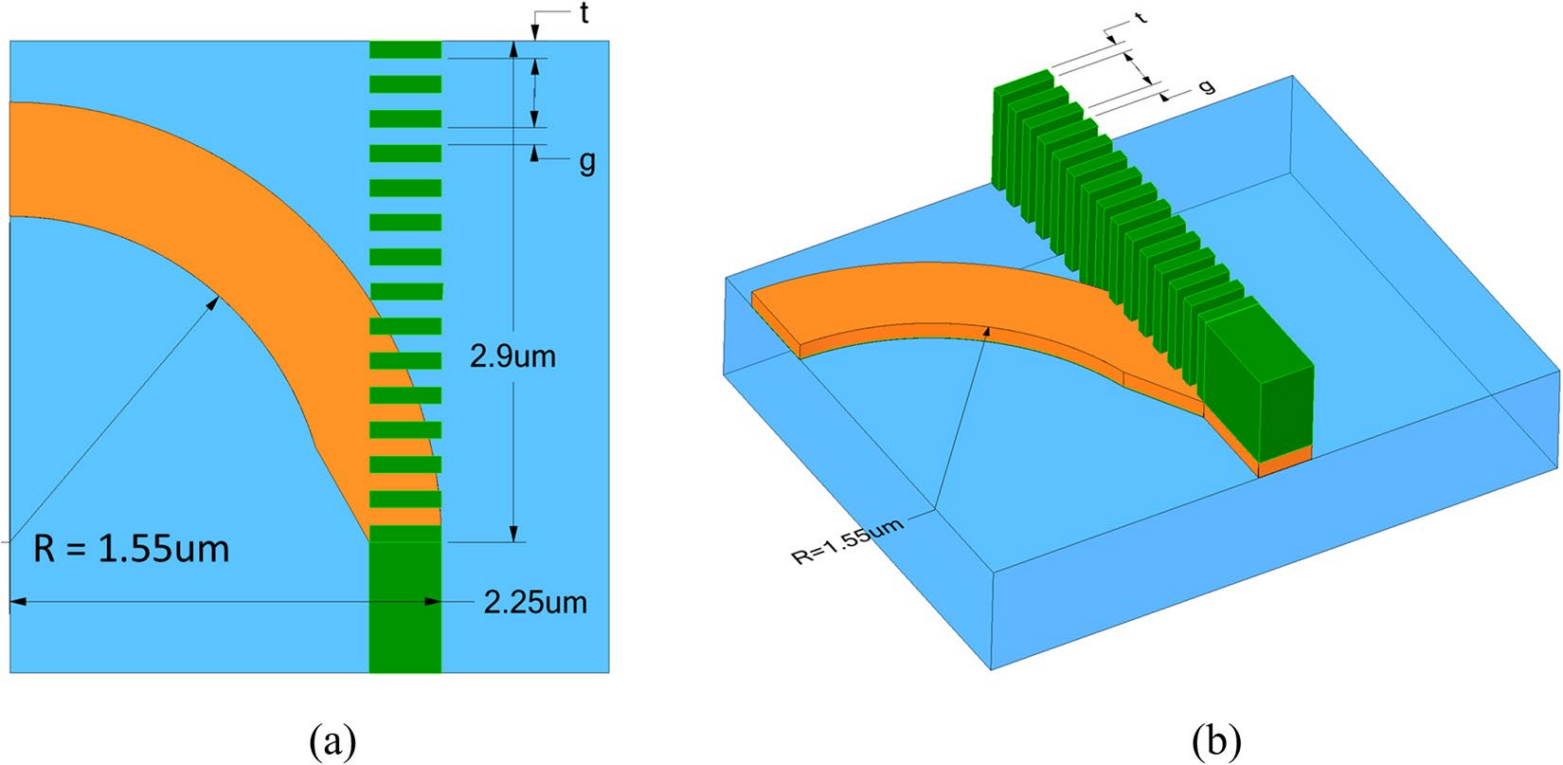
(**a**) Top view and (**b**) 3D schematic diagram of the proposed 3D PBS, including the SWG structure.

To significantly improve the PERs, we have modified the Si_3_N_4_ strip waveguide by using a SWG structure, as shown in Fig. 6, where we have adopted the dimension *R* = 1550 nm to preserve the performance shown in Fig. 5. The SWG structure consists of 14.5 grating pairs of pitch Λ (15 Si_3_N_4_ elements and 14 air gaps), consisting of the alternative materials Si_3_N_4_, of thickness t, and air, of thickness g. The proposed PBS has the dimensions 2.9 μm (length) × 2.25 μm (width).

**Figure 7:**
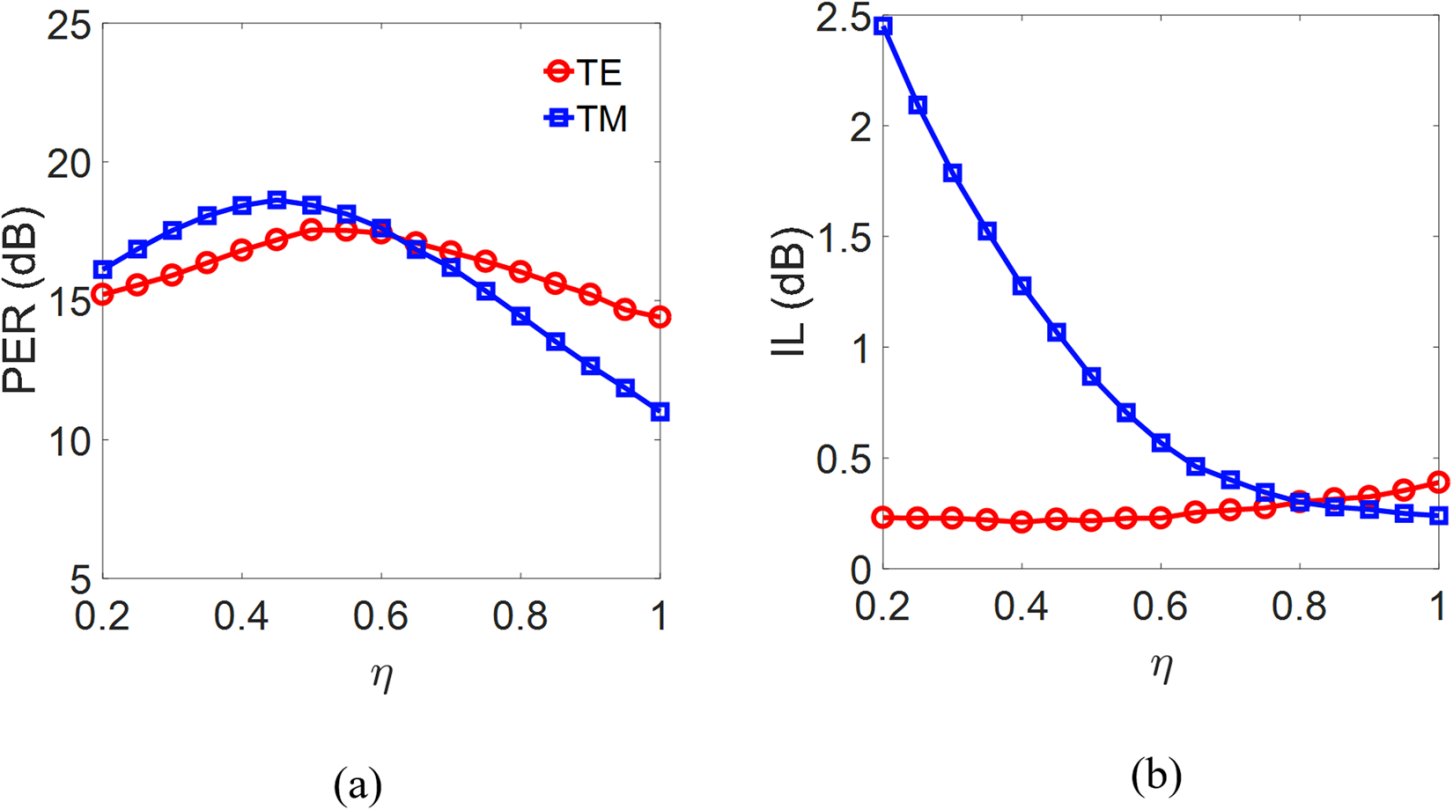
(**a**) PER and (**b**) IL of the TE and TM modes versus the duty cycle *η* of the subwavelength Si_3_N_4_ grating waveguide.

We know that light propagating in a grating structure (lengthwise periodic) behaves like electrons propagating in a periodic crystal. Therefore, it can be described as a Bloch mode, and its effective index *n*_B_ can be estimated from the following formula^21^:

1 $${n}_{B}^{2}\approx \eta {n}_{S{i}_{3}{N}_{4}}^{2}+(1-\eta ){n}_{air}^{2},$$

where *η* denotes the duty cycle of the grating structure. To operate in the subwavelength regime—so that the structure behaves as a conventional dielectric waveguide—we chose the condition *Λ* < *λ* / 2*n*_B_. In this work, to obtain the condition 1 < *n*_B_ < 2 from Eq. (1), the condition *Λ* < 387.5 nm must be satisfied at the wavelength *λ* = 1.55 μm. To alleviate the fabrication requirements and suppress diffraction, we chose *Λ* = 200 nm for operation in the subwavelength regime. The calculated PERs and ILs of the two modes as functions of *η* are shown in Fig. 7(a,b), respectively. The results show that the PERs of the two modes are significantly improved compared to that of the Si_3_N_4_ strip waveguide (i.e., to a grating structure with *η* = 1). When *η* = 0.55, the PERs of the TE and TM modes are 17.53 and 18.12 dB, respectively, and the ILs are 0.22 dB for the TE mode and 0.71 dB for the TM mode. Except for moderately scarifying the IL of the TM mode from 0.28 dB (the transmission is 93.7%) to 0.71 dB (the transmission is 84.9%), the proposed SWG structure considerably outperforms the Si_3_N_4_ strip waveguide structure for improving the PERs of the TE (from 15.35 to 17.53 dB) and TM (from 11.11 to 18.12 dB) modes, and the IL (from 0.48 to 0.22 dB) of the TE mode.

**Figure 8:**
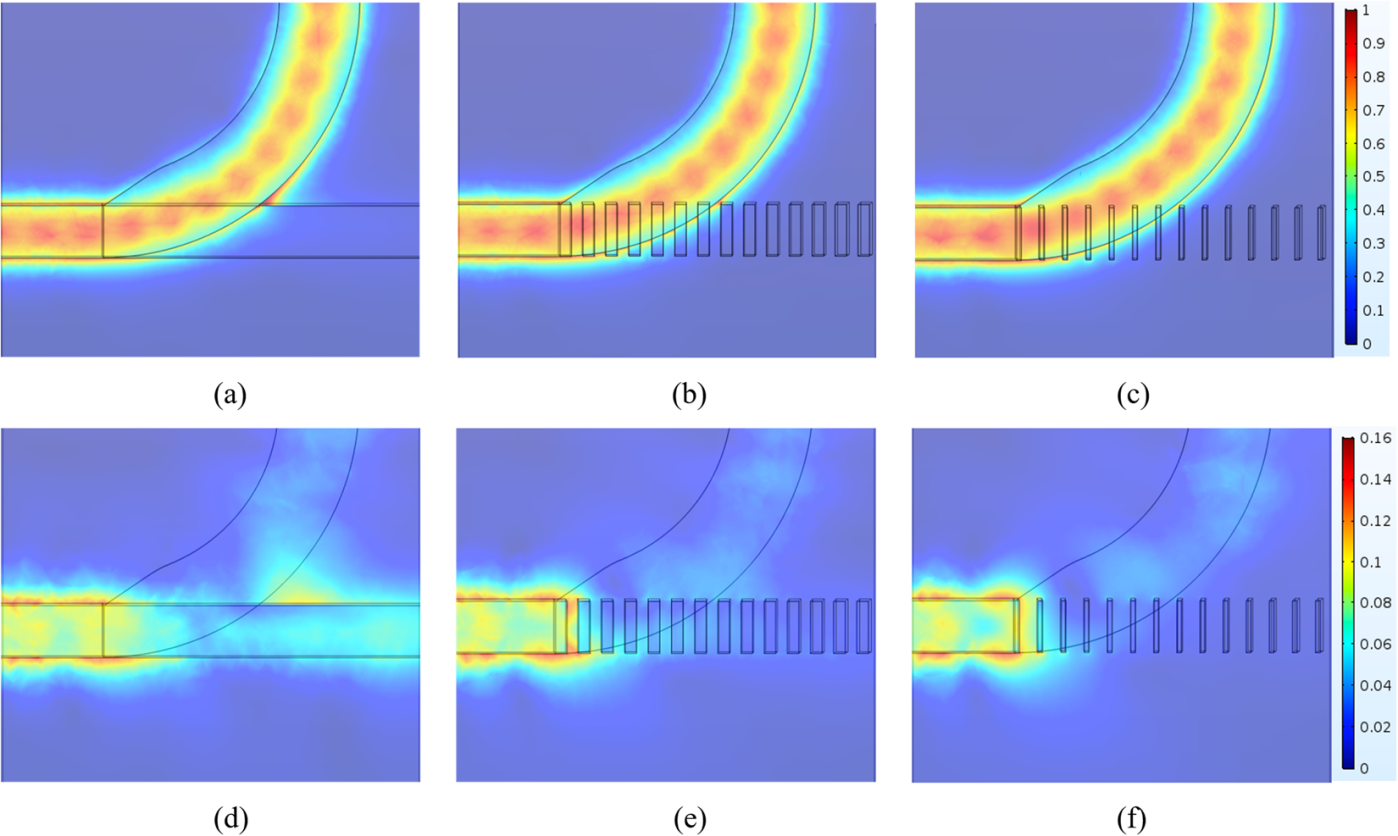
Field evolution |E| of the TE mode at the height *y* = *h*_Si_/2 for (**a**) *η* = 1, (**b**) *η* = 0.55, and (**c**) *η* = 0.2; and at the height *y* = (*h*_Si_ + *h*_Si3N4_)/2 for (**d**) *η* = 1, (**e**) *η* = 0.55, and (**f**) *η* = 0.2, where *y* = 0 is the bottom of the Si layer.

**Figure 9:**
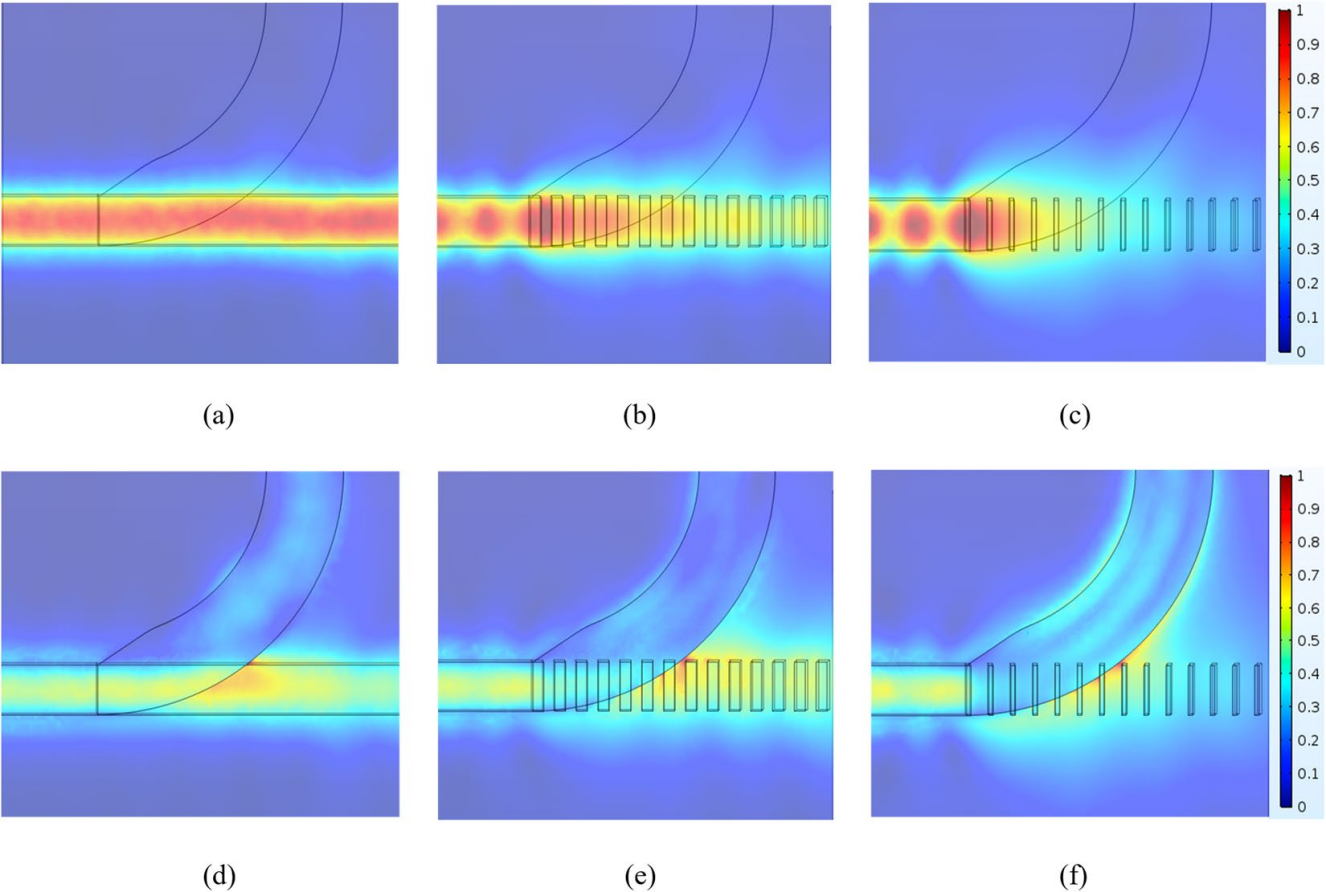
Field evolution |E| of the TM mode at the height *y* = *h*_Si_/2 for (**a**) *η* = 1, (**b**) *η* = 0.55, and (**c**) *η* = 0.2; and at the height *y* = (*h*_Si_ + *h*_Si3N4_)/2 for (**d**) *η* = 1, (**e**) *η* = 0.55, and (**f**) *η* = 0.2, where *y* = 0 is the bottom of the Si layer.

To understand these significant improvements in the PERs, we consider the principle of the periodic segmented waveguide structure^30–32^, noting that the waveguide dimensions Λ and η determine its modal characteristics. The device can be approximated by an equivalent strip waveguide with a refractive-index step given by^30,33^

2 $${\rm{\Delta }}{n}_{eq}=\eta \,{\rm{\Delta }}n$$

where Δ*n*_eq_ is the equivalent refractive-index step between the “composite core” and the cladding, and Δ*n* (=*n*_Si3N4_ – *n*_air_ = 1) is the refractive-index step between the original core and the cladding. From Eq. (4), the refractive-index of the equivalent Si_3_N_4_ strip waveguide is *n*_eq_ = 1 + *η* for the condition 1 < *n*_eq_ < 2. To explain clearly the significantly improved performance of the Si_3_N_4_ grating structure, we show in Figs 8 and 9, respectively, the mode field evolutions of the TE and TM modes at different positions.

**Figure 10:**
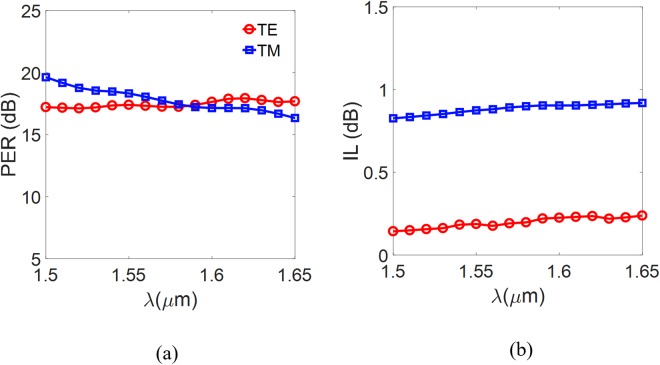
(**a**) PER and (**b**) IL of the Si_3_N_4_ grating structure, as functions of the operating wavelength λ.

In Fig. 8(a–f), we show the TE field distributions at the heights *h*_Si_/2 and (*h*_Si_ + *h*_Si3N4_)/2, respectively, for various values of *η*. Similarly, the TM field distributions at the heights *h*_Si_/2 and (*h*_Si_ + *h*_Si3N4_)/2 are shown in Fig. 9(a–f), respectively. Figure 8(a–c) show that the ILs of the TE modes vary only slightly with *η*. In contrast, the ILs of the TM modes vary significantly as *η* decreases, because of looser mode confinement, as shown in Fig. 9(a–c). For the PER of the TM mode, we find that decreasing *η* makes the effective index of the Si_3_N_4_ grating structure smaller for the TM mode, resulting in a larger effective index difference between the TE and TM modes. The effective refractive indices are *n*_TE_ = 1.9763 and *n*_TE_ = 2.1904 at the input and output ports, respectively. Because of the greater mismatch of the effective indices, it is more difficult for the TE power to be coupled into the TM grating waveguide, as shown in Fig. 8(d–f), (note that the maximum of the normalized electric field |E| is only 0.16), thus significantly increasing the PER of the TM mode. In particular, the optimal PER of the TM mode is reached around *η* = 0.45. This is because the transmission of the TM mode decreases for *η* smaller than 0.45, compensating for the lower TE power coupling, as shown in Fig. 9(a–c). In contrast to the PER of the TE mode, decreasing *η* decreases (increases) the TM power coupling to the curved Si waveguide, as shown in Fig. 9(d,e) [Fig. 9(e,f)], resulting in the optimal PER of the TE mode occurring around *η* = 0.55. To study the spectral response of the device, we have analyzed the PERs and ILs as functions of the operating wavelength *λ* at the condition of *η* = 0.55. Taking into account the material dispersion^29^, we show the calculated PERs and ILs in Fig. 10(a,b). The results show that the proposed PBS can be operated over a broad bandwidth of 150 nm, with PER > 17 dB for the TE mode and>16 dB for the TM mode, and with IL < 0.3 dB for the TE mode and <0.9 dB for the TM mode. When the device is operated over the narrower bandwidth from 1500 nm to 1600 nm, the PERs of the two modes are both greater than 17 dB. The results show that the designed PBS is wavelength-insensitive, due to avoidance of the phase-matching conditions and precise coupling that are required in DC- and MMI-based PBSs.

**Figure 11:**
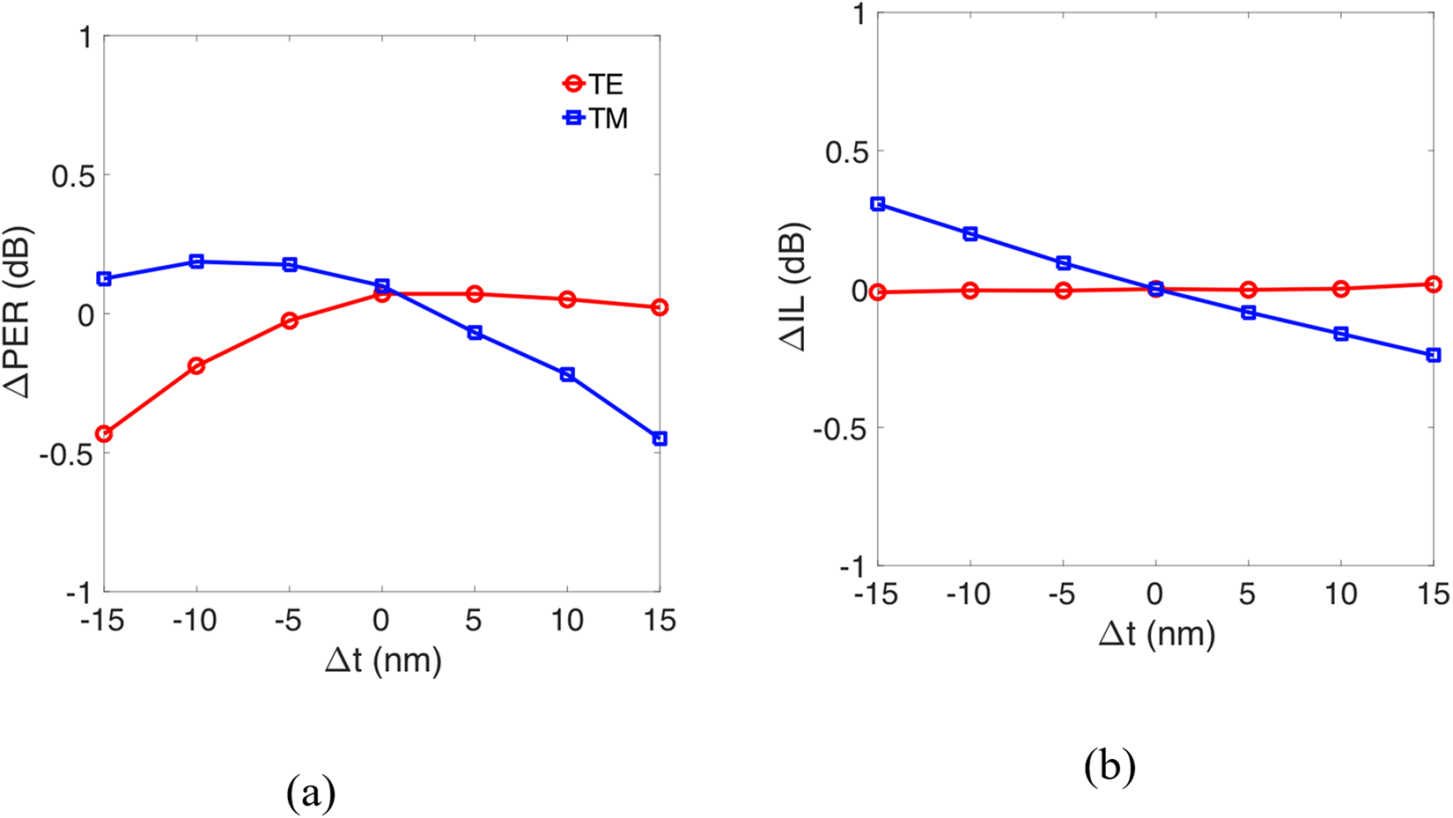
Variations of (**a**) Polarization extinction ratio (PER), ΔPER and (**b**) insertion loss (IL), ΔIL of proposed PBS with grating structure versus variation of t, Δt the thickness of the alternative materials Si_3_N_4_.

Finally, the fabrication tolerance was investigated to inspect the geometric parameters that affect the performance of the proposed PBS. We considered relatively smaller geometries such as the thicknesses of the alternative materials Si_3_N_4_, of thickness t and the Si layer *h*_Si_. From the analyzed results, we find that the degradations of the PER and IL with variations in t were all within ± 0.5 dB as shown in Fig. 11(a,b), where Δt = 0 denotes the condition of t = 110 nm, even when the parameter was varied by up to ±15 nm. The results confirm that the moderate variation of the thickness of the grating elements only slightly affects the performances of the proposed PBS.

**Figure 12:**
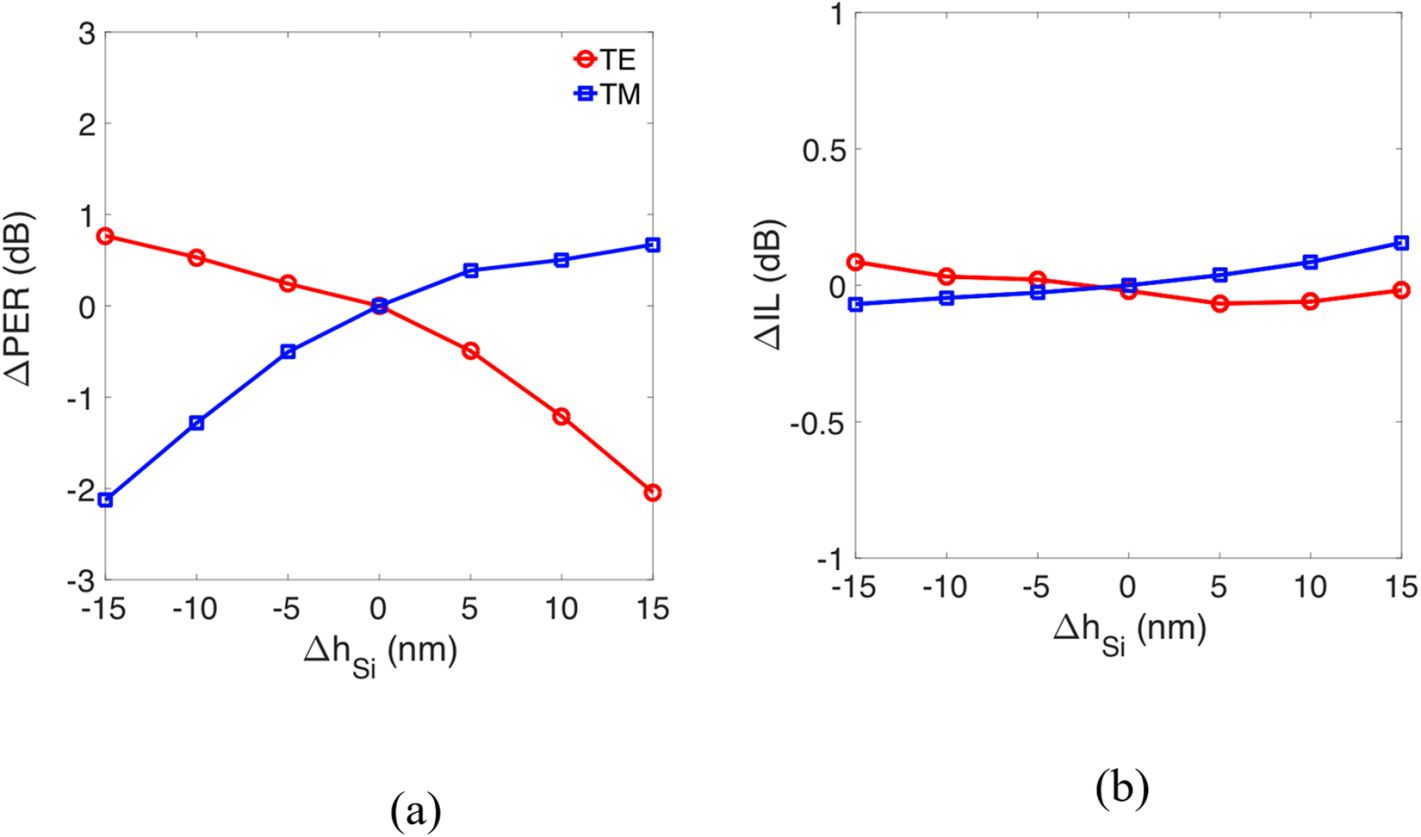
Variations of (**a**) Polarization extinction ratio (PER), ΔPER and (**b**) insertion loss (IL), ΔIL of proposed PBS with grating structure versus variation of *h*_Si_, Δ*h*_Si_ he thickness of the Si layer.

Next, we analyzed the effect of varying the thickness of the Si layer *h*_Si_. We find that the degradations of the PER and IL with variations in *h*_Si_ were within ±2 dB as shown in Fig. 12(a,b), where Δ*h*_Si_ = 0 denotes the condition of *h*_Si_ = 140 nm, when the parameter was varied by up to ±15 nm.

The calculated result shows that precisely controlling the thickness of the Si layer is the most essential for the reported PBS structure. This is because decreasing the thickness of the Si layer leads to more power fraction of the TE mode extending out of the Si region. Therefore, more TE power is coupled into the TM channel while it propagates along a curved waveguide, reducing the PER of the TM mode. In contrast, the PER of the TE mode is improved slightly because the larger difference of the mode profiles between the TE and TM modes further reducing the TM power coupled into the TE channel as shown in Fig. 12(a). Fortunately, the roughness of deposited thickness of the Si layer can be controlled precisely under 10 nm by the modern fabricated technique such as low-pressure, chemically-vapor-deposited (LPCVD)^34^. As a result, the degradation of the PER can be reduced to within ±1 dB. In summary, the numerical results demonstrate that the present PBS has a high fabrication tolerance.

For clearly comparing the merits of various PBSs, we summarize some structures in Table 1. It can be seen that the structures [8] and [9] with easy and moderate fabricating difficulties, respectively, obtain smaller footprint than the proposed PBS, however, the ILs (~3 dB) of [8] are fairly high due to use of copper nanorod and the PERs (~10 dB) of [9] are low due to much overlapping of the two polarizations. Comparing with the bandwidth of this work with the range of ~150 nm, the structures [8] and [9] are capable of operating in the ranges of ~80 and 280 nm, respectively. Overall, the footprint (2.9 × 2.25 μm^2^) of the proposed PBS with moderate fabricating difficulty is slightly larger than the two structures [8], [9] but both of the PERs (>17 dB and >16 dB for the TE and TM modes, respectively) and ILs (<0.3 dB and <0.9 dB for the TE and TM modes, respectively) are better than those of structures [8] and [9]. Considering the performances of PER and IL, MMI coupler on an InP substrate^12^ provides the best values in Table 1, however; the footprint (950 × 13 μm^2^) is considerably large and is detrimental to form highly dense photonic integrated circuits (PICs). In summary, comparing to other structures in Table 1, the proposed PBS possesses relatively balancing merits.

**Table 1:** Comparisons of various polarization beam splitters.

Structures	Footprint (μm^2^) (Length × Width)	PER(dB)	IL (dB)	Bandwidth (nm)	Fabricating difficulty
Mode-evolution-based [4]	200 × 2.15	>22	—	~300	Moderate
Hybrid plasmonic and silicon nanowire waveguides [5]	3.7 × 1.9	>12	<1	~120	Easy
Asymmetrical bent DC [6]	9.5 × 2.5	>10	<0.1 (TE), <1 (TM)	~200	Moderate
Asymmetrical multimode slot waveguide [7]	8.3 × 4.0	>9 (TE), >17 (TM)	<4 (TE)<2 (TM)	100	Complicated
Two-mode interference coupler [9]	13.4 × 1.8	>15	<0.8 (TE), <1.7 (TM)	50	Easy
MIM inserted into MMI coupler [10]	44 × 4	~12.55	1.1 (TE), 0.9 (TM)	—	Easy
MMI coupler with a hybrid plasmonic waveguide [11]	2.5 × 1.8	~10	<1	80	Easy
MMI coupler on InP substrate [12]	950 × 13	~34	<0.8	22	Easy
ALIG with an asymmetric MMI section [19]	4.8 × 1.6	>24 (TE), >16 (TM)	<0.4	100	Easy
DC with a SWG-based structure [23]	21 × 1.83	>10	<1	175	Moderate
ALIG with a SWG-based coupler structure [24]	7.2 × 3	>15	<0.35	110	Complicated
This work	2.9 × 2.25	>17 (TE), >16 (TM)	<0.3 (TE), <0.9 (TM)	150	Moderate

DC: directional coupler; MIM: metal-insulator-metal; MMI: multimode interference; ALIG: augmented low-index guiding; SWG: subwavelength grating.

In conclusion, we have reported the design of an ultra-compact PBS based on an ALIG structure and a SWG structure, which can confine the TE and TM modes in high-index Si and low-index Si_3_N_4_ regions, respectively. Instead of using the MMI structure reported in previous work, which results in PBS dimensions of 4.8 μm (length) × 1.6 μm (width), the proposed PBS separates the TE mode by adopting a gradually curved Si waveguide. It separates the TM mode by using a Si_3_N_4_ grating waveguide with an optimal profile. This design significantly shrinks the dimensions to 2.9 μm (length) × 2.25 μm (width) while maintaining PERs about 18 dB for both modes and ILs of ~0.22 dB (~0.71 dB) for the TE (TM) mode at the wavelength of 1550 nm. Over the broadband from *λ* = 1500 to 1600 nm, the proposed PBS has PERs of >17 dB for both the TE and TM polarizations, demonstrating that the proposed device is wavelength-insensitive. This work points to a new route for the design of various photonic integrated circuits by utilizing flexible arrangements of the elements in a grating structure.

## Methods

In this study, to evaluate the transmission characteristics of a PBS, we calculated the PER and IL of a specific mode as defined in Eqs (3) and (4), respectively:

3 $$PE{R}_{TE(TM)}=10{\mathrm{log}}_{10}({P}_{1\,(2)}^{TE(TM)}/{P}_{1\,(2)}^{TM(TE)}),$$

and

4 $$I{L}_{TE(TM)}=-\,10{\mathrm{log}}_{10}({P}_{1(2)}^{TE(TM)}/{P}_{{\rm{input}}}^{TE(TM)}),$$

where ${P}_{i}^{{\rm{TE}}}\,{\rm{and}}\,{P}_{i}^{{\rm{TM}}}$ denote the mode power at output port *i* (*i* = 1 or 2) for the TE and TM modes, respectively.
